# High-Accuracy 3D Optical Profilometry for Analysis of Surface Condition of Modern Circulated Coins

**DOI:** 10.3390/ma13235371

**Published:** 2020-11-26

**Authors:** Wojciech Kapłonek, Tadeusz Mikolajczyk, Danil Yurievich Pimenov, Munish Kumar Gupta, Mozammel Mia, Shubham Sharma, Karali Patra, Marzena Sutowska

**Affiliations:** 1 Department of Production Engineering, Faculty of Mechanical Engineering, Koszalin University of Technology, Racławicka 15-17, 75-620 Koszalin, Poland; wojciech.kaplonek@tu.koszalin.pl (W.K.); marzena.sutowska@tu.koszalin.pl (M.S.); 2 Department of Production Engineering, UTP University of Science and Technology, Al. prof. S. Kaliskiego 7, 85-796 Bydgoszcz, Poland; tami@utp.edu.pl; 3 Department of Automated Mechanical Engineering, South Ural State University, Lenin Prosp. 76, 454080 Chelyabinsk, Russia; danil_u@rambler.ru (D.Y.P.); munishguptanit@gmail.com (M.K.G.); kpatra@iitp.ac.in (K.P.); 4 Key Laboratory of High Efficiency and Clean Mechanical Manufacture, Ministry of Education, School of Mechanical Engineering, Shandong University, Jinan 250061, China; 5 Department of Mechanical Engineering, Imperial College London, Exhibition Rd., South Kensington, London SW7 2AZ, UK; 6 Department of Mechanical Engineering, IK Gujral Punjab Technical University, Jalandhar-Kapurthala Road, Kapurthala 144603, India; shubham543sharma@gmail.com; 7 Department of Mechanical Engineering, Indian Institute of Technology Patna, Patna 800013, India

**Keywords:** circulated coins, surface condition, optical methods, measurements and analysis

## Abstract

The article shows that noncontact measurement techniques can be an important support to X-ray-based methods when examining the surface condition of modern circulated coins. The forms and degrees of wear of such coins, affecting their utility values, qualifying them as a legal tender in a given country, can be measured and analyzed, among other things, using advanced high-accuracy optical profilometry methods. The authors presented four analyses carried out for reverses and obverses of round coins (1 zloty, 1 franc, 50 bani, 5 pens) characterized by different degrees of surface wear. All of the coins were measured using 3D optical profilometers (Talysurf CLI 2000 and S neox) representing two generations of these types of systems. The obtained results confirm the validity of the applied high-accuracy measurement systems in conjunction with dedicated software in the presented applications. Examples of the analyses carried out can be a significant source of information on the condition of coins in the context of maintaining their functional properties (selection of appropriate wear–resistant alloys and correctness of the production process).

## 1. Introduction

Coins, as cash marks bearing the issuers’ marks and used as legal tenders in countries, have long been an interesting and intriguing object of scientific research. They exist, similar to banknotes, in many world monetary systems. Coin production has not changed significantly for centuries. Today, their production process has been greatly improved—production time has been shortened, and the use of new, promising materials has positively influenced their quality and performance characteristics, as reported by de Toit et al. and Mendoza-López et al. in their works [1,2], but the very idea of production has remained the same—coins were and are embossed.

**Figure 1:**
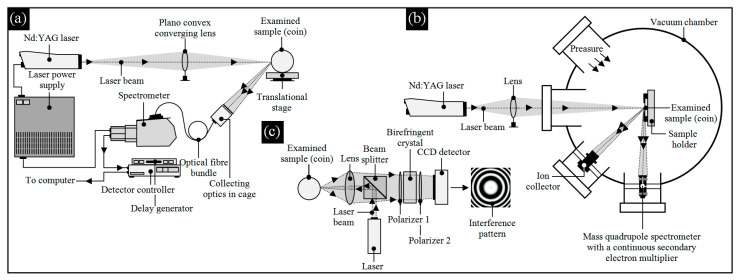
Selected experimental setups used for analyzing of the ancient coins: (**a**) laser-induced breakdown spectroscopy (LIMB), presented in the work of Awasthi et al. [14] (coins collected from the G.R. Sharma Memorial Museum, University of Allahabad, India, dating back to VI Cent. A.D.); (**b**) laser ablation coupled with mass quadrupole spectrometry (LAMQS), presented in the work of Torrisi et al. [8] (Mediterranean basin bronze coins, dating back to the II–X Cent. A.D.); (**c**) high-resolution laser microprofilometry using conoscopic holography (CH), presented in the work of Spagnolo et al. [43] (ancient Roman coins, dating back to the I Cent. A.D.).

Ancient coins, widely described in the works of Dow [3] and Metcalf [4], obtained by archaeological methods are extremely rich sources of historical information. Such information is usually received based on analyses of the chemical composition, corrosion products, surface morphology, microstructure, and physical properties, and then correlated with known manufacturing processes, sources of raw materials, and finally, geographical distribution of ancient mints. The above analyses (depending on the characteristics of the coin, complexity of the process of preparing the coin for research, and intensity of the research program) are carried out using many advanced observation measurement X-ray-based methods. Among them, the most significant methods are those using diffractometry, spectroscopy, electron microscopy, and computed tomography. A multitude of these methods and their variations, metrological characteristics, and specificity of application (not every technique is appropriate for the assessment of a given coin or group of coins) allow for the perception of the complexity of carried-out analyses and the real difficulty of assessing ancient coins. This is confirmed by numerous scientific papers from this research area published over the last several years. A representative review of the above X-ray-based methods and their application with adequate references are given in Table 1, and in Figure 1, the selected X-ray- and optical-based experimental setups carrying out the ancient coin analyses are presented.

**Table 1:** Selected X-ray-based methods used for the analysis of ancient coins.

Application	Method	References
Analysis of chemical composition and microstructure	EDS (EDX)	[5] ^1^, [6] ^2^
	EDXRF	[7] ^4^, [8,9,10,11]
	GRT	[12]
	LAMQS	[8]
	LIBS	[13,14]
	ND	[15,16]
	PIXE	[6] ^2^, [17] ^3^, [18], [6] ^2^, [19], [20] ^3^, [21,22,23,24,25,26]
	RBS	[25]
	XPS	[27] ^4^, [28]
	XRD	[5] ^1^, [7] ^4^, [16,28]
	XRF	[12,13,29,30], [31] ^3^
	SRXRF	[6] ^2^, [19] ^4^
	SEM	[32] ^3^, [33,34]
	TEM	[33,35]
Analysis of the corrosion products	EDS (EDX)	[36] ^1,5^
	PIXE	[37,38] ^3^
	XPS	[36] ^1,5^
	XRD	[36] ^1,5^, [38] ^3^, [39] ^4^
	XRF	[38] ^3^
Reconstruction	CT	[40,41,42]

^1^ Authors also used a combination of scanning electron microscopy (SEM) and optical microscopy (OM); ^2^ authors also used SEM; ^3^ based on chemical composition/corrosion products, authors carried out an analysis of the authenticity of the coins; ^4^ for a study of the homogeneity/heterogeneity/ presence of surface enrichments, authors also used a combination of SEM and energy-dispersive X-ray spectrometry (EDS/EDX); ^5^ authors also used electron microprobe analysis (EMPA).

Coins in their modern form are mainly circulation coins. Similarly, coins such as antique ones produced of metals and their alloys are subject to the wear phenomenon. It is a natural consequence of circulation. Wear occurs with varying intensities over the entire surface of a given coin in a whole or fragmentary manner as a result of a range of physical and chemical interactions. Everyday handling of coins (e.g., tossing and/or removing them from cash registers; storing them in wallets, pockets, and bags; accidentally dropping them on a hard surface; etc.) causes small losses of material. Therefore, apart from their purely mint (uncirculated) conditions, from the time they are put into circulation, coins are subject to wear. This process can be divided into several phases:

- Phase I: Slight wear (the coin entering the circulation begins to show signs of manipulation, abrasion, or slight wear, which can be stated after a visual analysis of changes in fragments of the surface texture and differences in the sharpness of fine elements (mint mark, motto, etc.), as well as slight differences in their color).
- Phase II: Average wear (the coin in circulation, after a period of time, begins to show signs of medium wear. It can be stated after a visual analysis of the design’s highest points, which begin to lose their sharpness and slightly round or flatten, while fine elements close together begin to connect).
- Phase III: Extensive wear (the coin in circulation, after a long time, begins to show signs of extensive wear. It can be stated after a visual analysis of the design sharpness. High points begin to connect with the next lower elements of the design. After flattening the protective rim, the entire surface begins to flatten—most of the details connect with itself or partially with the surface).
- Phase IV: Critical wear (the coin in circulation, after a long time, begins to show signs of critical wear. This phase causes the completely flattening of the protective rim, design, and all other elements of the coin. Both the obverse and reverse are difficult to identify. The coin cannot fulfill the tender function and must be withdrawn from circulation).

**Figure 2:**
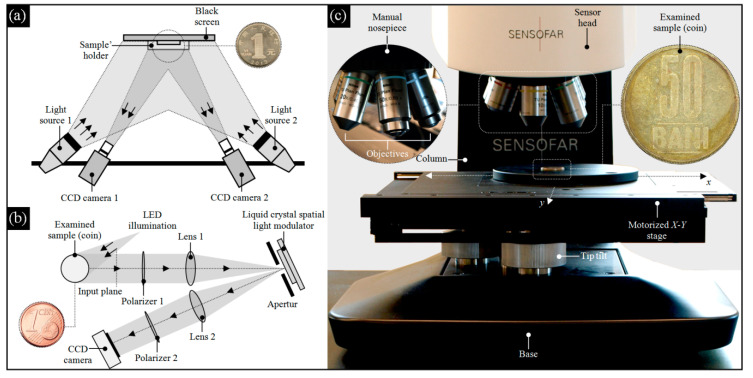
Selected experimental setups used for analyzing modern coins: (**a**) three-dimensional digital image correlation (3D-DIC), presented in the work Yan et al. [63] (1 yuan coin); (**b**) computational shear interferometry (CSI), shown in the work of Falldorf et al. [64] (1 euro cent coin); (**c**) confocal laser scanning microscopy (CLSM) (50 bani coin).

Similar to ancient coins, modern coins are subjected to numerous studies that allow the determination of their chemical composition, as presented in the work of Roumie et al. [44], for high accuracy and surface micromorphology characterization, as reported in the work of Papp and Kovacs [45], and allow the localization of surface defects (stains), as presented in the work of Corregidor et al. [46]. The above studies are carried out using advanced X-ray-based observation measurement methods [47], which are often supported by other noncontact techniques. A significant number of such techniques are optical profilometry methods [48]. These methods and their many variants allow for comprehensive studies of coins with the use of advanced geometric (dimensional shape) analyses and surface texture measurements (mainly roughness). Modern 3D optical profilometers are excellent platforms for this type of study. These platforms offer, among others, the possibility of integration of noncontact and high-accuracy measurement methods in one instrument, wide measurement range, short time of surface scanning, wide range of analysis, and surface visualization modes available in specialized software cooperating with the measurement instrument. A representative review of the above methods and their applications with adequate references are given in Table 2, whereas in Figure 2, selected optical-based experimental setups for analyses of modern coins are presented.

**Table 2:** Selected optical profilometry-based methods used for the analysis of modern coins.

Application	Method	References
Measurements of shape and surface profile (2D)	FP	[49]
	FP + SP	[50]
	FP + PS	[51]
	FP + PS + TPU + DIA	[52]
	MI	[53]
Measurements of shape (3D)	SL	[54]
Measurements of surface profile and topography (3D)	FP	[54,55,56,57]
	FVM	[58]
	CLSM	[59]
	CS	[60,61]
	SL	[62]

To familiarize readers with selected issues related to the assessment of the microtopography of circulated coins (Section 2.1) with various degrees of surface wear using optical profilometry methods, the authors of this article carried out experimental studies. During these experiments, measurement systems (Section 2.2) representing two generations of high-accuracy 3D optical profilometers were used. The main part of the work focused on the presentation of selected analyses of circulated coins representing various degrees of surface wear. In Section 3, four of such analyses were discussed. Each of them consisted of information on the type of measurement system used, parameters of the measurement process, and selected results of the analyses carried out, along with a detailed description and interpretation.

## 2. Experimental Studies

### 2.1. Selection and Characteristics of the Coins

For the experimental studies, a set of six round-shaped coins in uncirculated (one coin used as reference) and circulated (five coins used in main studies) conditions was selected. Each of the coins had individual physical (composition, geometrical dimensions) and visual (relief) features. A main criterion for selecting the coins was their visually observed surface condition (forms and wear degrees). The general characteristics of the analyzed modern coins are presented in Table 3.

Before starting the measurements, the surfaces of the coins were cleaned with undiluted acetone, then washed in water with the addition of mild detergent (soap), and carefully dried with compressed air.

### 2.2. Characteristics of Observation Measurement Systems

To carry out accurate measurements and imaging of the surfaces of small-size objects (coins), the authors selected appropriate metrological systems. Two types of profilometric-based measurement systems using optical methods were used in the experimental studies. 

The first of the systems was the multisensory 3D optical profilometer Talyscan CLI 2000 (Taylor- Hobson, Leicester, UK). The system was characterized by the following properties: capacity: 200 × 200 × 200 mm; traverse length: 200 mm at resolution 0.5 μm; measuring speed: 0.5, 1.5, 10, 15, and 30 mm/s; and positioning: 30 mm/s. During the experiments, the measuring head of the instrument was equipped with the confocal chromatic (CLA) sensor RB-800 (scanning frequency: 5000 Hz; measuring range: 0.8 mm; resolution (vertical): 0.025 μm; speed: 30 mm/s). The Talyscan CLI 2000 2.6 software was used for realizing the measurement process, whereas the TalyMap Silver 4.1 software using Mountains Technology^®^ (Digital Surf, Besançon, France) was utilized for the analysis and visualization of the surface microtopography. A detailed description of this older-generation instrument (2003–2004) was presented in the work of Kapłonek et al. [60], whereas its exemplary applications were given by Fan et al. [65], Saremi-Yarahmadi et al. [66], Beamud et al. [67], and Genna et al. [68].

The second of the systems was the 3D optical profilometer S neox (Sensofar Metrology, Terrassa, Spain). This new-generation instrument used three main optical methods (confocal, interferometry, and focus variation) and was characterized by the following properties: capacity: 700 × 600 × 40 mm; maximum vertical scanning range: 20, 100, 10 (interferometry), and 37 mm (confocal, focus variation); resolution and linearity (*z*-axis): 2 nm; and <0.5 μm/mm linear stage. The motorized revolving nosepiece of the S neox was equipped with five Brightfield type TU Plan Fluor EPI (semiapochromat) 5×, 10×, 20×, and 50× and TU Plan Apo EPI (apochromat) 150× microscopic lenses (Nikon Corp., Tokyo, Japan). The SensoSCAN 2.0 software provided the correct course of the measurement process, whereas SensoMAP Premium software using Mountains Technology^®^ (Digital Surf, Besançon, France) was used for advanced analysis and visualization of the surface microtopography. A comprehensive description of this measurement system was given in the work of Artigas et al. [69], whereas a review of its applications can be found in the works of Ding et al. [70], Leksycki and Królczyk [71], Leksycki et al. [72], and Tato et al. [73].

The abovementioned measurement systems were supported by the 3D laser microscope LEXT OLS4000 (Olympus Corp., Shinjuku, Tokyo, Japan), bench-type multisensory coordinate measuring machine VideoCheck^®^ IP 250 (Werth Messtechnik, Gießen, Germany), and digital microscope Omni Core (Ash Technologies Ltd., Kildare, Ireland).

## 3. Results and Discussion

### 3.1. Comparative Analysis of Uncirculated/Circulated 1 Złoty (Obverse) Coin’s Surface Condition

In many cases, circulated coins’ wear is determined by advanced instrumental observations (microscopy) or accurate measurements (profilometry) of their obverse or reverse, often using uncirculated coins as reference. In this subsection, such type of comparative analysis was presented.

**Figure 3:**
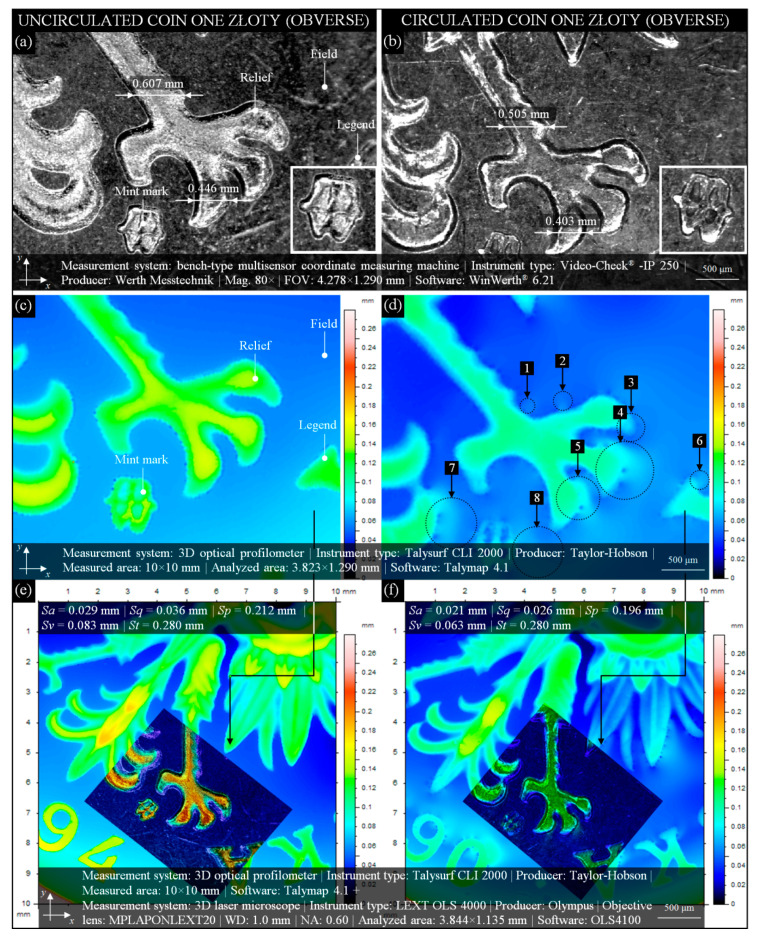
Selected results of observation and analysis of the surface condition of modern Polish uncirculated and circulated 1 złoty coin: images of the obverse of (**a**) uncirculated and (**b**) circulated 1 złoty coin issued in 1994 and 1990, respectively, acquired by bench-type multisensory coordinate measuring machine VideoCheck^®^ IP 250 (Werth Messtechnik, Gießen, Germany) with characteristic elements and dimensions corresponding to images from Figure 1a,b, the 2D height map (indexed colors) obtained by the 3D optical profilometer Talysurf CLI 2000 (Taylor-Hobson, Leicester, Great Britain) for the obverse of (**c**) uncirculated and (**d**) circulated 1 złoty coin with the visible worn surface of the field as well as relief, mint mark, and legend; image fusion of a vast fragment (10 × 10 mm) of (**e**) uncirculated and (**f**) circulated coin presented in the form of a 2D height map (indexed colors) with a fragment (3.844 × 1.135 mm) obtained by the 3D laser microscope LEXT OLS4000 (Olympus Corp., Shinjuku, Tokyo, Japan) in the form of a 2D pseudo-color height map.

Figure 3 depicts a modern Polish 1 złoty coin (alloy: Cu_75_Ni_25_; edge: alternately smooth and serrated; diameter: 23 mm; thickness: 1.70 mm; weight: 5 g). Figure 3a presents an image obtained by the bench-type multisensory coordinate measuring machine (CMM) VideoCheck^®^ IP 250 for a fragment (4.278 × 1.290 mm) of an uncirculated coin issued in 1994. Uncirculation, in this case, caused the coin’s surface to present an excellent condition. Obverse (engraver: S. Wątróbska-Frindt) side elements—a fragment of relief, mint mark (enlarged miniature at bottom right), and legend—are clear and sharp. Additionally, the spatial nature of the above elements (they are convex) is visible.

An image from Figure 3a corresponding to the 2D height map (indexed colors) of a fragment (3.823 × 1.290 mm) of the same coin obtained by the 3D optical profilometer Talysurf CLI 2000 is presented in Figure 3c. The height of the field is in the range from ~0.06 to 0.08 mm, whereas the height of the elements on it is ~0.08 to ~0.16 mm. This shows the relatively high differentiation of the height observed on a surface of such uncirculated coin. The circulated 1 złoty coin issued in 1990 is presented in Figure 3b. The differences in geometric dimensions (from ~0.04 to 0.1 mm) of the selected elements of this coin as compared with the uncirculated coin (Figure 3a) indicate a significant average wear (Phase II). The relief is clearly flattened, as are the mint mark (enlarged miniature at bottom right) and legend. The height change of the elements can also be analyzed on the 2D height map (Figure 3d), where a slight surface wear, worn areas of the coin (marked as 1–8), is presented. Using measurement data obtained by the 3D optical profilometer Talysurf CLI 2000 and 3D laser microscope LEXT OLS4000, the image fusion presented in Figure 3e,f was generated. A vast fragment (10 × 10 mm) of the uncirculated (Figure 3e) and circulated (Figure 3f) coins was presented in the form of a 2D height map (indexed colors) with a fragment (3.844 × 1.135 mm) of a 2D pseudo-color height map. This combination perfectly reflects the differences in the heights of the depicted relief elements (eagle claws) of the uncirculated (clearly convex) and circulated (clearly flattened) coins and strongly corresponds to previous analyses (Figure 3c,d). Additionally, Figure 3e,f provides the values of selected amplitude (surface) parameters, *Sa*, *Sq*, *Sp*, *Sv*, and *St,* included in the ISO 25178-2:2012 standard [74] and EUR 15178 EN report [75]. These parameters are correlated and have a significant impact on the operating properties. The essential parameters from this group—*Sa* (arithmetic mean deviation of the surface) and *Sq* (root-mean-square deviation of the surface)—represent an overall measure of the surface texture. The *Sq* parameter is usually used to characterize optical surfaces (more smooth) and *Sa* machined surfaces (more irregular). The use of these parameters was dictated by the similar characteristics of the analyzed surfaces—uncirculated coin (more smooth) and circulated coin (more irregular). The values of these parameters showed slight differences in the range from 0.008 mm (*Sa*) to 0.010 mm (*Sq*). The additional parameters *Sp* (maximum height of summits) and *Sv* (maximum depth of valleys) showed slightly larger differences in the range from 0.016 mm (*Sp*) to 0.030 mm (*Sv*), whereas the value of the parameter *St* (total height of the surface) for both surfaces was the same and amounted to 0.280 mm. The analysis of the values of selected amplitude (surface) parameters allows for the conclusion that, generally, the values obtained for the circulated coin were ~7% to ~27% lower than the parameters obtained for the uncirculated coin. The surface of the circulated coin was, therefore, distinguished by the average wear characteristic described in Introduction Phase II.

### 3.2. Analysis of the Circulated 1 Franc and 50 Bani Coins’ Edge Surface Condition

It seems that the obverse and reverse of the coin are the elements most often exposed to wear. Another equally important element that is also subject to the wear process is the edge. There are different types of edges, most often plain (smooth) or patterned (mostly reeded and lettered). Edges usually have a decorative function, but also prevent coin clipping and counterfeiting.

In this subsection, studies carried out on the edges of two modern circulation coins—French 1 franc and Romanian 50 bani—were presented. Each of the analyses included the following elements: general view of the coin, a fragment of the coin’s edge, and based on marks on the edge of the area of interest (AOI), 2D height map, surface microtopography, and close-up view with calculated values of selected surface texture parameters and extracted single surface profiles.

**Figure 4:**
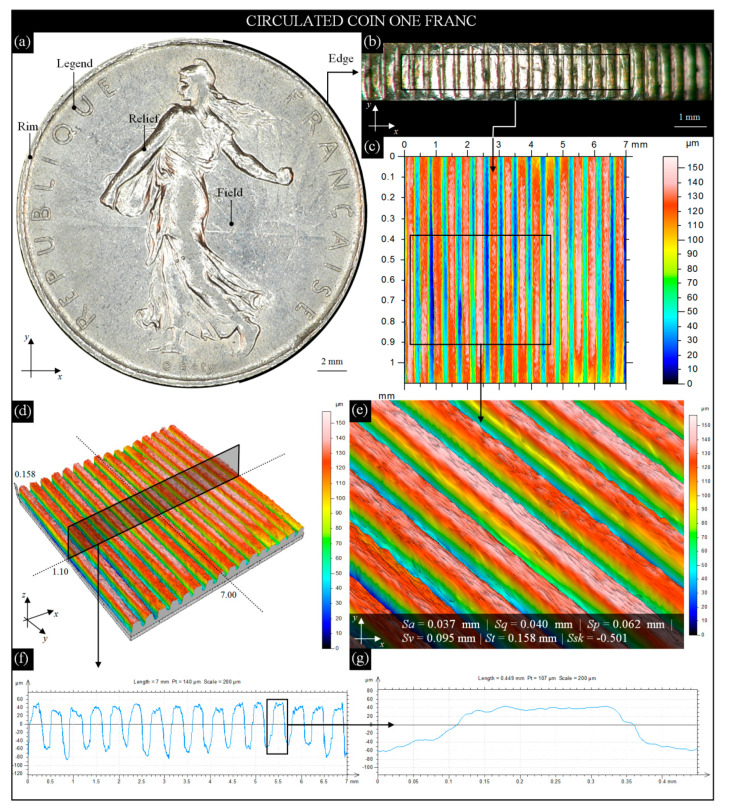
Selected results of observation and analysis of the surface condition of modern circulated French 1 franc coin obtained by the digital microscope Omni Core (Ash Technologies Ltd., Kildare, Ireland) and 3D optical profilometer S neox (Sensofar Metrology, Terrassa, Spain): (**a**) general view of coin obverse; (**b**) an image (10.93 × 1.79 mm) of a fragment of the coin’s reeded edge; (**c**) a 2D height map (indexed colors) of the AOI (7.11 × 1.10 mm) from Figure 4b; (**d**) surface microtopography (7.11 × 1.10 × 0.15 mm); (**e**) close-up view (4.49 × 0.51 mm) of the reeded edge with calculated amplitude (surface) parameters; (**f**) extracted from Figure 4d, a single surface profile with visible grooves; (**g**) extracted from Figure 4f and enlarged image of a single groove.

The image of the obverse (engraver: L. O. Roty) of a modern French 1 franc coin (alloy: Ni; edge: reeded; diameter: 24 mm; thickness: 1.79 mm; weight: 6 g) acquired by the digital microscope Omni Core (lens: +5; WD: 200 mm; magnification: 45×) is presented in Figure 4a with characteristic elements (the *Sower*, designed by O. Roty in 1900, a national emblem of the French Republic). 

For a fragment of the right side of the coin, mounted vertically in a holder, an image (10.93 × 1.79 mm) of its reeded edge (Figure 4b) was acquired. The condition of the edge surface showed minimal wear on the upper and lower parts, whereas relatively higher wear occurred on the grooves. They were slightly flattened, and some were vertically deformed. Despite this, the wear was considered to be average (Phase II) as convex of the grooves was visible, which were usually strongly flattened for extensively worn coins (Phase III).

On the image of the reeded edge (Figure 4b), the AOI (7.11 × 1.10 mm) was marked, for which measurements by the 3D optical profilometer S neox were carried out. The obtained results in the form of a 2D height map (indexed colors) and surface microtopography (area of the topography (axes *x*, *y*, *z*): 7.00 × 1.10 × 0.15 mm; number of profile points (axis *x*): 7001; distance between profile points (axis *x*): 1 μm; number of profiles (axis *y*): 158; distance between profile points (axis *y*): 7 μm) are presented in Figure 4c,d. On the 2D height map (indexed colors), an AOI (4.49 × 0.51 mm) was marked.

For this AOI, a close-up view of the reeded edge was extracted (Figure 4e). Additionally, the values of the selected amplitude (surface) parameters, *Sa*, *Sq*, *Sp*, *Sv*, *St*, and *Ssk*, were added. From surface microtopography (Figure 4d), a single surface profile (type: west–east; surface size: 7.00 × 1.00 mm; profile size: 7.00 mm (7001 points)) was extracted (Figure 4f). The profile consisted of 16 slightly worn grooves on the edge, and one of them was additionally enlarged and is shown in Figure 4g. Analysis of data from the 3D optical profilometer revealed apart from the wear on the outer surfaces of the grooves, the wear also occurred in the spaces between the subsequent grooves (Figure 4c). With an average depth of ~0.050 mm, there were numerous valleys even below 0.060 mm (Figure 4f). The *Ssk* (skewness of the height distribution) parameter, defined as the degree of symmetry of the surface heights to the mean plane, was used in the presented analysis. In this case, the value of *Ssk* is > 0, which indicates the predominance of peaks composing the surface.

**Figure 5:**
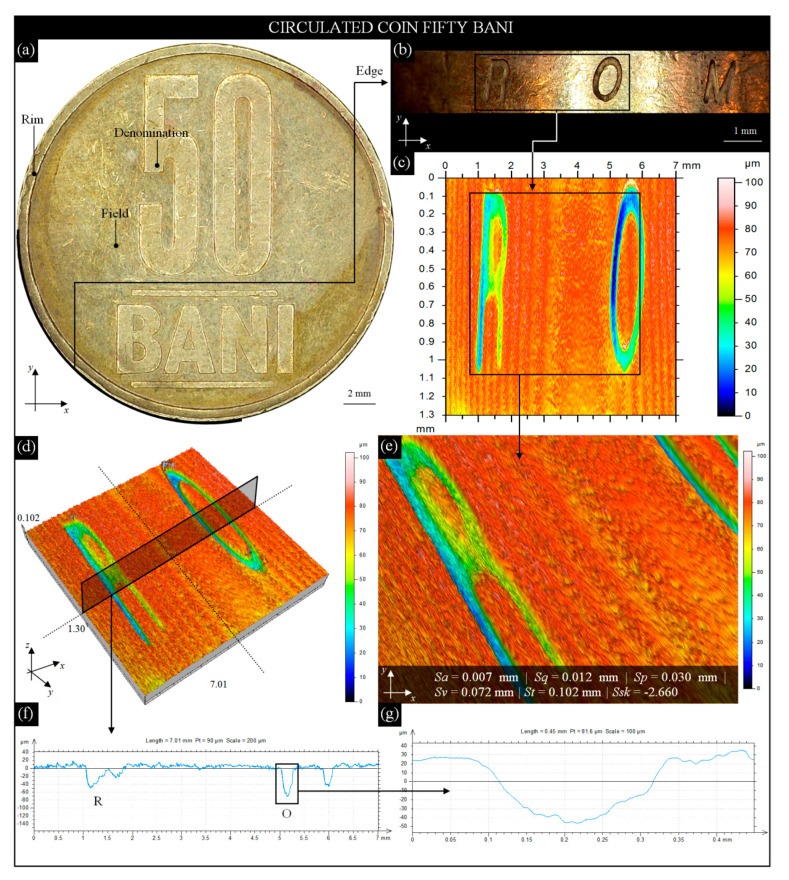
Selected results of observation and analysis of the surface condition of modern Romanian circulated 50 bani coin obtained by the digital microscope Omni Core (Ash Technologies Ltd., Kildare, Ireland) and 3D optical profilometer S neox (Sensofar Metrology, Terrassa, Spain): (**a**) general view of coin reverse; (**b**) image (11.42 × 1.90 mm) of a fragment of the coin’s smooth and lettered edge; (**c**) 2D height map (indexed colors) of the AOI (7.01 × 1.30 mm) from Figure 4b; (**d**) surface microtopography (7.01 × 1.30 × 0.10 mm); (**e**) close-up view (5.10 × 1.00 mm) of the smooth and lettered edge with calculated amplitude (surface) parameters; (**f**) extracted from Figure 4d, a single surface profile with deformed left side of the letter O; (**g**) extracted from Figure 5f and enlarged letter O.

The studies prepared for the second coin was identical and included elements listed at the beginning of this subsection. The image in Figure 5a presents a reverse of a modern Romanian 50 bani coin (alloy: Cu_80_Zn_15_Ni_5_, edge: smooth and lettered; diameter: 23.75 mm; thickness: 1.90 mm; weight: 6.10 g) acquired by the digital microscope Omni Core (lens: +5; WD: 200 mm; magnification: 45×) with the characteristic element (lettering 50 BANI). For a fragment of the left side of the coin, mounted vertically in a holder, an image (11.42 × 1.90 mm) of its smooth and lettered edge (Figure 5b) was acquired. The condition of the edge shows average wear on the smooth surface (numerous scratches and slightly lost material) and around the letters R O M. The strongest deformation was observed in the lower part of the letter O. Circulation of this coin caused a visible average/extensive (locally) wear of the surface. Its intensity allows the conclusion that the coin surface wear process is between Phase II and Phase III. On the image of the smooth and lettered edge (Figure 5b), the AOI (7.01 × 1.30 mm) was marked, for which the optical measurements by the 3D optical profilometer S neox were carried out. This high-accuracy system allowed for obtaining a set of measurement data (Figure 5c,d) in the same form as previously presented for the 1 franc coin—the 2D height map (indexed colors) and surface microtopography (area of topography (axes *x*, *y*, *z*): 7.01 × 1.130 × 0.10 mm; number of profile points (axis *x*): 7001; distance between profile points (axis *x*): 1 μm; number of profiles (axis *y*): 187; distance between profile points (axis *y*): 7 μm). From the marked AOI in Figure 5c (5.10 × 1.00 mm), a fragment of the surface in an area of letter R and O was extracted. This close-up view of the smooth surface of the bani coin with calculated amplitude (surface) parameters *Sa*, *Sq*, *Sp*, *Sv*, *St*, and *Ssk* is presented in Figure 5e. In a similar way as in previous analysis, from surface microtopography (Figure 5d), a single surface profile (type: west–east, surface size: 7.01 × 1.30 mm; profile size: 7.01 mm (7001 points)) was extracted (Figure 5f). The profile passes through two letters of the edge—R and O. The deformation of the letter O (Figure 5g) is visible in the form of its depression being more than twice as large (~0.072 mm) on the left side relative to the right side (~0.039 mm). The values of the amplitude (surface) parameters *Sa*, *Sq*, *Sp*, *Sv*, *St*, and *Ssk*, calculated for circulated 50 bani coins, were on average more than 50% lower than for the circulated 1 franc coin. In the case of the skewness of the height distribution value determined for the 1 franc coin, *Ssk* > 0 indicates the predominance of peaks composing the surface. Visual analysis and values above the parameters confirmed the generally worse surface condition of this coin, with the process being between Phase II and Phase III.

### 3.3. Comparative Analysis of the Circulated 50 Bani (Reverse) Coin’s Surface Condition

In Section 3.1, a comparative analysis of the obverses of two 1 złoty Polish coins was presented. The circulated coin representing average degree of wear (Phase II) was compared with the reference uncirculated coin. A similar analysis is presented in this section, although two of the same face value circulated coins, but with totally different surface conditions, were compared in this case. The results, showing the differences in the denomination state in the central part of the field, were presented in the form of individual surface profiles extracted from 2D height maps (indexed colors).

**Figure 6:**
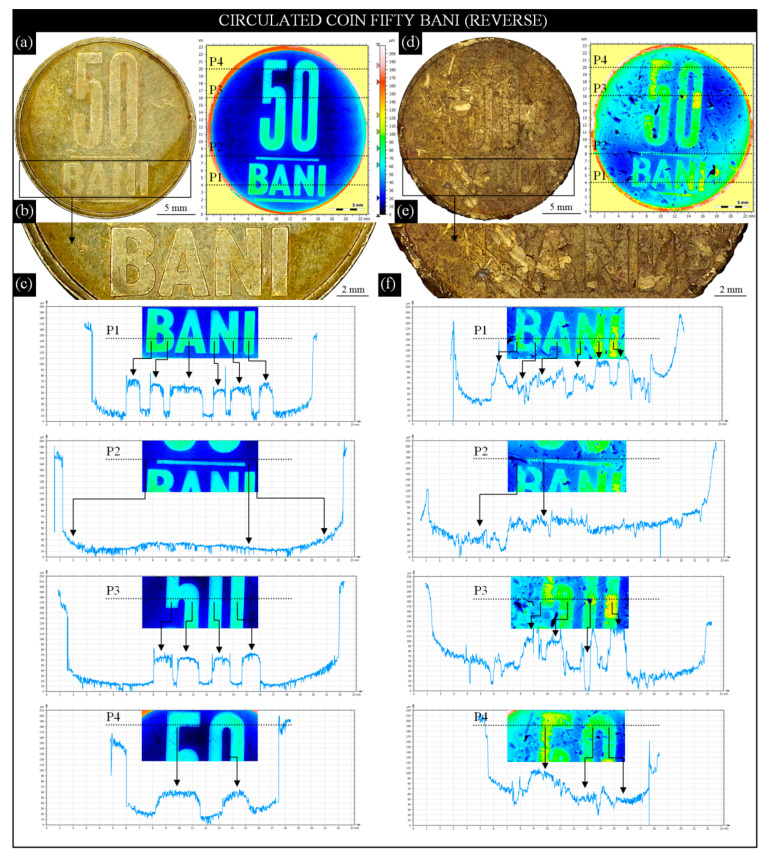
Selected results of observation and analysis of the surface condition of modern Romanian circu- lated 50 bani coins obtained by the digital microscope Omni Core (Ash Technologies Ltd., Kildare, Irela- nd) and 3D optical profilometer S neox (Sensofar Metrology, Terrassa, Spain): (**a**–**d**) general view of the coins (left: in condition between average and extensive wear (Phase II/III); right: in condition representing critical wear (Phase IV)) with a corresponding 2D height map (indexed colors); (**b**–**e**) AOIs (22.70 × 4.91 mm) extracted from Figure 6a–d presenting enlarged lettering B A N I for various conditions of surface wear; (**c**–**f**) surface profiles (P1–4 mm, P2–8, P3–16, and P4–20 mm) extracted from each of the coins.

For analysis, two modern Romanian 50 bani coins (alloy: Cu_80_Zn_15_Ni_5_; edge: smooth and lettered; diameter: 23.75 mm; thickness: 1.90 mm; weight: 6.10 g) were prepared. The condition of the coins was selected for analysis in such a way as to obtain visible differences during the visual observation. The first coin (Figure 6a) represented a condition between average (Phase II) and extensive wear (Phase III), whereas the second (Figure 6d) represented critical wear (Phase IV).

With these two images acquired by the digital microscope Omni Core (lens: +5; WD: 200 mm; magnification: 45×), 2D height maps (indexed colors) corresponded. The maps were obtained for the entire surface of both coins (area (axes *x*, *y*): 30.30 × 27.30 mm; number of profile points (axis *x*): 11,755; distance between profile points (axis *x*): 2.58 μm; number of profiles (axis *y*): 10,587; distance between profile points (axis *y*): 2.58 μm by the 3D optical profilometer S neox. Figure 6b shows an AOI (22.70 × 4.91 mm) extracted from Figure 6a presenting an enlarged lettering of B A N I. The letters in the field of this coin are slightly distorted at the ends, and there are also visible slight scratches on their surface. Despite these, the lettering is legible. On the abovementioned 2D height map (Figure 6a), a set of four surface profiles (type: west–east; surface size: 23.00 × ~0.22 mm) was marked and presented in Figure 6c. Each of the profiles were determined for the characteristic element of the denomination: P1–for lettering B A N I, P2—for the area of the field above the line, P3—for the lower part of the number 50, P4—for the upper part of the number 50. All the described elements were well recognizable on the profiles (they were additionally marked with arrows), which confirms the relatively good (in this context) condition of this part of the coin. Figure 6d–f presents the same analyses as above, prepared for the seconds of the analyzed coins. They include a general view of the coin with a 2D height map (indexed colors) (Figure 6d); AOI (22.70 × 4.91 mm) extracted from Figure 6d presenting enlarged lettering B A N I (Figure 6e); and a set of four surface profiles (type: west–east, surface size: 23.00 × ~0.22 mm) (Figure 6f). Visual analysis of the second circulation coin allows us to state a high degree of its wear corresponding to the critical wear (Phase IV). The protective rim was flattened also, and numerous material losses were visible. The edge was strongly deformed. The lettering B A N I was highly flattened and difficult to identify. The field was characterized by numerous scratches, abrasions, and material losses. The 2D height map (indexed colors) revealed details of the above defects. A clearly lower surface height was visible in the central region of the coin, while numerous losses of the material dominated around the central part. The study of the profiles (P1–P4) showed a large surface degradation in terms of height. The elements of the denomination were highly flattened and difficult to identify, which qualifies the coin for immediate withdrawal from circulation.

### 3.4. Analysis of the Circulated 5 Pence (Reverse) Coin’s Surface Condition 

The last analysis presented in this section aims to show the possibility of visualizing surfaces using the confocal fusion processing algorithm, combining measurement data from confocal and focus-variation images of the surface. This algorithm was widely described in the works by Artigas et al. [76] and Bermudez et al. [77], whereas selected examples of its application are given in the works of Flys et al. [78], Hatami et al. [79], and Maruda et al. [80].

**Figure 7:**
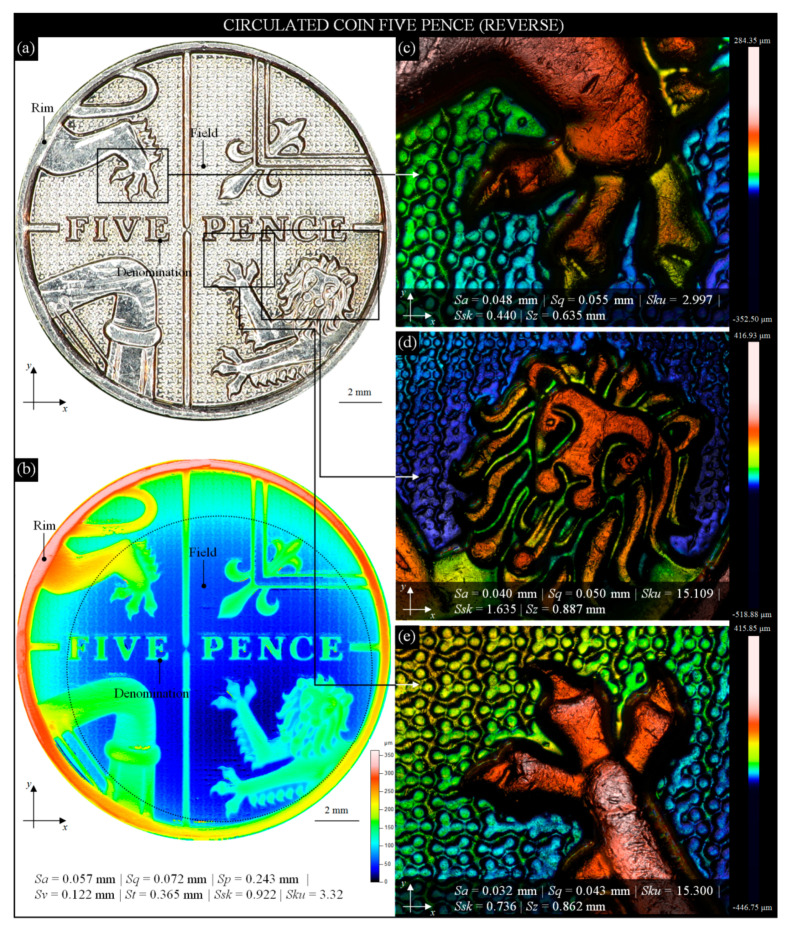
Selected results of observation and analysis of the surface condition of a modern British circulated 5 pence coin obtained by the digital microscope Omni Core (Ash Technologies Ltd., Kildare, Ireland), 3D optical profilometer Talysurf CLI 2000 (Taylor-Hobson, Leicester, Great Britain), and 3D optical profilometer S neox (Sensofar Metrology, Terrassa, Spain): (**a**) general view of coin reverse; (**b**) 2D height map (indexed colors) with calculated amplitude (surface) parameters; (**c**) extracted from Figure 1a, AOI (3.32 × 2.92 mm) depicting a lion claw (front foot); (**d**) extracted from Figure 7a, AOI (5.52 × 4.42 mm) depicting a lion head; (**e**) extracted from Figure 7a, AOI (3.32 × 2.92 mm) depicting a lion claw (rear foot).

The reverse image (engraver: M. Dent) of a modern British 5 pence coin (alloy: Ni-plated steel, edge: reeded; diameter: 18 mm; thickness: 1.89 mm; weight: 3.25 g) acquired by the digital microscope Omni Core (lens: +5; WD: 200 mm; magnification: 45×) is presented in Figure 7a with characteristic elements (royal shield of arms and the lettering FIVE PENCE in the central position). Using the 3D optical profilometer Talysurf CLI 2000, the entire coin was measured and is presented in Figure 7b in the form of a 2D height map (indexed colors). In the marked AOI (13.78 × 13.78 mm), typical for everyday handling of the coin, the relative slight wear (in terms of surface height) was clearly visible.

Based on the obtained measurement data, selected amplitude (surface) parameters *Sa*, *Sq*, *Sp*, *Sv*, *St*, *Ssk*, and *Sku* were calculated and presented below this figure. Analysis of their values shows that the overall wear of the coin’s surface is between slight (Phase I) and average (Phase II). Visualizations of selected fragments of the 5 pence coin using the confocal fusion processing algorithm based on mea- surement data obtained by the 3D optical profilometer S neox are presented in Figure 7c–e. Extracted from Figure 7a, the AOI (3.32 × 2.92 mm) depicting a lion claw (front foot) is shown in Figure 7c.

This high-resolution visualization (3665 × 2757 pixels) shows the structure of the coin in an area of relief and a fragment of the characteristic texture of the field. The next AOI (5.52 × 4.42 mm) represents a lion head (Figure 7d), and another AOI (3.32 × 2.92 mm) depicts a lion claw (rear foot) (Figure 7e). The above high-resolution visualizations were obtained by precise point-by-point scanning of the coin’s surface, and processing obtained in this way a single scan by smart confocal fusion algorithm. As a result, a set of surface topographies was created, which were stitched together (image stitching procedure), allowing to obtain an output large area topography. The great advantage of such topography was the fact that it retained a high-resolution and represented the same quality of the detail. This, in turn, allowed for the precise observation of various forms and intensities of wear in selected areas of the coin. For all of the AOIs, an individual set of selected amplitude (surface) parameters, *Sa*, *Sq*, *St*, *Ssk*, and *Sku*, was added. The differences between the parameters were relatively small and resulted mainly from the height of the elements of a given relief as well as local, more intensive wear of the surface. The values of the *Ssk* parameter showed the predominance of peaks, whereas the values of the *Sku* parameter indicated lack of inordinately high peaks or deep valleys in the measured areas.

## 4. Conclusions

This article was an attempt to familiarize the readers with issues related to the analysis of circulation coins in the context of observation, visualization, and measurement of various forms of wear occurring on their surfaces. Additionally, the authors’ intention was to present the use of advanced methods based on optical profilometry in this specific type of applications. The obtained results of measurements and studies allowed the authors to draw the following detailed conclusions:

1. Most of the analyses of modern circulation coins focus on the precise determination of their chemical composition. In this case, modern varieties of X-ray-based observation measurement methods, such as EDS (EDX), EDXRF, LAMQS, and, PIXE, were used (Section 1). To extend the results of the spectroscopic examinations, 2D/3D dimensional-shape measurements (macroscale) and surface texture measurements (micro scale) were carried out. Additionally, the authors located and recognized the surface defects. Modern measurement methods, such as CLSM, FVM, and advanced variants of interferometry, using optical profilometry, prove to be helpful in these activities.
2. The authors of the article showed that optical profilometry could be successfully used in the analysis of the surface condition of modern circulation coins representing Phases I–IV of surface wear. The measurement capabilities of the systems used (Section 2.1) turned out to be sufficient for the needs of the carried-out experimental tests. The undoubted advantages of the 3D optical profilometers used were relatively quick measurement time, noncontact method of assessment, high resolution and measurement range, integration of (optical) measurement methods in one instrument, and advanced processing and visualization of measurement data. In more complex cases, this type of tests may be carried out with the use of additional measuring instruments or more specialized computer software—it depends on the application.
3. The analyses presented in the article (Section 3) are general and illustrative—they are shown against the background of hardware and software capabilities. Analyses of the surface condition of coins can be much more advanced (comprehensive) or strictly focused on a specific feature of the assessed surface. For example, it can relate with mint-made errors (errors from a three fundamental groups, including blank planchet, fundamental die-setting, and broadstrike) generated during the coin manufacturing process. Such errors occurring during the minting practice must be located and adequately analyzed. Another interesting area is concerning the analyses carried out to establish the authenticity of a given coin or group of coins. Such activities usually refer to the antique coin(s), but in justified cases, they may also refer to coins illegally released into circulation.
4. Special computer software (especially based on Mountains Technology^®^) provided significant support in the carried-out analyses. Its universal character and the number of implemented functions it performed were useful in characterizing coin surfaces. In case of a need to generate the output large area topography, the image stitching procedure was conducive.
5. In the authors’ opinion, the subject discussed in this article is extremely interesting and has a chance to be further developed. There is a plan to carry out a more detailed study (e.g., analysis of surface wear in the context of changes of coin relief height). Such changes are extremely important because critical wear makes it impossible to use a given coin as a legal tender and, as a result, causes its withdrawal from circulation. The authors also plan to carry out a wider research program using advanced X-ray-based observation measurement methods supported by noncontact techniques (optical profilometry), where issues regarding the influence of elemental composition on the wear process will be considered.

## Figures and Tables

**Table 3:** Characteristics of the modern coins used in experimental studies.

Coin Value	Country	Type	Years	Composition (Alloy)	Diameter (mm)	Thickness (mm)	Weight (g)
1 złoty	Poland	Standard circulation coin	1990–2016	Cu_75_Ni_25_	23.00	1.70	5.00
1 franc	France		1959–2001	Ni	24.00	1.79	6.00
50 bani	Romania		2005–2017	Cu_80_Zn_15_Ni_5_	23.75	1.90	6.10
5 pence	Great Britain		2011–2015	Ni-plated steel	18.00	1.89	3.25

## Nomenclature

3D-DIC	Three-dimensional digital image correlation (3D-DIC)
AOI	Area of interest
CH	Conoscopic holography
CS	Confocal sensor
CSI	Computational shear interferometry
CLA	Chromatic light aberration phenomenon
CLSM	Confocal laser scanning microscopy
CMM	Coordinate measuring machine
CT	X-ray computed tomography
DIA	Digital image acquisition
EDS	Energy-dispersive X-ray spectrometry
EDXRF	Energy-dispersive X-ray fluorescence spectrometry
EMA	Electron microprobe analysis
FP	Fringe projection
FVM	Focus variation microscopy
GRT	Gamma-ray transmission
LAMQS	Laser ablation coupled to mass quadrupole spectrometry
LIBS	Laser-induced breakdown spectroscopy
MI	Moiré interferometry
ND	Neutron diffraction
OM	Optical microscopy
PIXE	Particle (proton)-induced X-ray emission
PS	Phase stepping
RBS	Rutherford backscattering spectrometry
XPS	X-ray photoelectron spectroscopy
XRD	X-ray diffraction
SEM	Scanning electron microscopy
SL	Structured light
SP	Speckle projection
SRXRF	Synchrotron radiation X-ray fluorescence spectrometry
TEM	Transmission electron microscopy
TPU	Temporal phase unwrapping
WD	Working distance, mm
*Sa*	Arithmetic mean deviation of the surface, µm
*Sku*	Kurtosis of the height distribution, -
*Sp*	Maximum height of summits, µm
*Sq*	Root-mean-square deviation of the surface, µm
*Ssk*	Skewness of the height distribution, -
*St*	Total height of the surface, µm
*Sv*	Maximum depth of valleys, µm

## Glossary

Circulated—a term used to refer to a coin characterized by various conditions of surface wear (from slight wear to critical wear).
Circulation—a term used to refer to a coin that is currently or was in the past in circulation as a means of payment (money).
Denomination—a value assigned by a government to a given coin.
Design—motif, pattern, or emblem used in the coin.
Edge—the third side of a coin, containing reeds, lettering, or other ornaments. The edge can also be plain.
Field—a flat (or slightly curved) area of a coin with no emblem or inscription.
Motto—word, sentence, or phrase inscribed on a coin to express a guiding national principle.
Obverse—front or head side of the coin.
Relief—any element of a coin’s design that is raised above the field. The opposite of relief is incuse.
Reverse—back or tail side of the coin.
Rim—raised portion of the design along the edge that protects the coin from wear.
Uncirculated—theoretically, a coin that has never circulated and thus retains all of its original mint conditions (a coin without wear with excellent surface condition).
